# Adolescent Female Text Messaging Preferences to Prevent Pregnancy After an Emergency Department Visit: A Qualitative Analysis

**DOI:** 10.2196/jmir.6324

**Published:** 2016-09-29

**Authors:** Lauren Stephanie Chernick, Rebecca Schnall, Melissa S Stockwell, Paula M Castaño, Tracy Higgins, Carolyn Westhoff, John Santelli, Peter S Dayan

**Affiliations:** ^1^ Division of Pediatric Emergency Medicine Department of Pediatrics Columbia University Medical Center New York, NY United States; ^2^ Department of Nursing Columbia University Medical Center New York, NY United States; ^3^ Division of Child and Adolescent Health Department of Pediatrics Columbia University Medical Center New York, NY United States; ^4^ Department of Population and Family Health Mailman School of Public Health New York, NY United States; ^5^ Department of Obstetrics-Gynecology Columbia University Medical Center New York, NY United States

**Keywords:** pregnancy in adolescence, emergency medicine, text messaging, reproductive health, contraception, preventive medicine

## Abstract

**Background:**

Over 15 million adolescents use the emergency department (ED) each year in the United States. Adolescent females who use the ED for medical care have been found to be at high risk for unintended pregnancy. Given that adolescents represent the largest users of text messaging and are receptive to receiving text messages related to their sexual health, the ED visit represents an opportunity for intervention.

**Objective:**

The aim of this qualitative study was to explore interest in and preferences for the content, frequency, and timing of an ED-based text message intervention to prevent pregnancy for adolescent females.

**Methods:**

We conducted semistructured, open-ended interviews in one urban ED in the United States with adolescent females aged 14-19 years. Eligible subjects were adolescents who were sexually active in the past 3 months, presented to the ED for a reproductive health complaint, owned a mobile phone, and did not use effective contraception. Using an interview guide, enrollment continued until saturation of key themes. The investigators designed sample text messages using the Health Beliefs Model and participants viewed these on a mobile phone. The team recorded, transcribed, and coded interviews based on thematic analysis using the qualitative analysis software NVivo and Excel.

**Results:**

Participants (n=14) were predominantly Hispanic (13/14; 93%), insured (13/14; 93%), ED users in the past year (12/14; 86%), and frequent text users (10/14; 71% had sent or received >30 texts per day). All were interested in receiving text messages from the ED about pregnancy prevention, favoring messages that were “brief,” “professional,” and “nonaccusatory.” Respondents favored texts with links to websites, repeated information regarding places to receive “confidential” care, and focused information on contraception options and misconceptions. Preferences for text message frequency varied from daily to monthly, with random hours of delivery to maintain “surprise.” No participant feared that text messages would violate her privacy.

**Conclusions:**

Adolescent female patients at high pregnancy risk are interested in ED-based pregnancy prevention provided by texting. Understanding preferences for the content, frequency, and timing of messages can guide in designing future interventions in the ED.

## Introduction

Approximately 15 million adolescents use an emergency department (ED) each year in the United States [1]. Adolescent females in the ED have a risk of pregnancy that is up to 3 times higher than the general population; this risk is associated with reduced contraception use and lack of a primary care doctor [2,3]. Despite female adolescents expressing an interest in learning about contraception while in the ED, current methods of referral to reproductive health preventive care services from the ED show limited success [4,5].

Mobile technology has the potential to play a role in the reproductive health among adolescents [6]. Text messaging is a fast, convenient, low-cost, and scalable way of sending information [7]. In the United States, nearly 3 quarters of adolescents have access to a mobile phone, with teen females sending and receiving an average of 4050 texts per month [7,8].

In the outpatient setting, the use of text messaging to improve adolescent reproductive health shows promise; however, data exploring pregnancy prevention interventions using text messages from the ED are limited [9]. Data are needed to understand adolescents’ preferences in order to develop an engaging and acceptable text messaging intervention [10]. Our objective was to study interest in and preferences for the content, structure, and timing of an ED-based text messaging pregnancy prevention intervention for adolescent females at high risk of pregnancy.

## Methods

We conducted semistructured interviews from June to October 2013 at an urban tertiary care pediatric ED. The Institutional Review Board approved the study with written informed consent from participants and a waiver of parental permission.

### Study Participants

We enrolled a convenience sample of females aged 14-19 years who presented to the ED. Eligibility required (1) being sexually active with a male partner in the past 3 months, (2) having a reproductive health complaint, and (3) being at high risk for pregnancy that is defined as nonuse of contraception at the last intercourse and currently not using any hormonal contraception or an intrauterine device. We excluded patients if pregnant, trying to become pregnant, too sick per the attending physician, cognitively impaired, in foster care or a ward of the state, or if they did not own a mobile phone. We enrolled only English-speaking patients as prior studies demonstrated that the adolescent Hispanic population in our ED is bilingual [2].

### Study Procedures

The research team identified potentially eligible patients using an electronic tracking board. If a patient met the inclusion criteria, the research team privately explained the study to the patient and obtained written consent. After obtaining consent, participants completed a paper-based questionnaire in the ED regarding demographics, access to care, sexual behaviors, and pregnancy intentions. One of the 2 trained interviewers (LC or RS) conducted and audio-recorded interviews in a private ED room. All interviews were then transcribed by a professional service.

### Interview Guide

The study team iteratively wrote the interview guide. Questions explored participants’ interest in receiving pregnancy prevention texts from the ED. Probes included preference for texting versus other forms of mobile technology (email, Internet, and phone calls) and concerns of confidentiality. Questions then focused on the preferred content, timing, and frequency of text messages. Participants viewed a mobile phone with a sample of 10-15 messages and provided their preferences regarding the content and structure as well as preferences for frequency and timing.

### Text Messages

Based on previous literature, we created text messages with both an educational component and action component [11]. The educational component, or “hooks”, was a motivational or informative statement such as a pregnancy prevention message or information on contraceptives. We used the Health Belief Model to design the message “hook” [12]. Each message addressed one of the following: perceived severity or susceptibility of being or becoming pregnant, perceived barriers or benefit of starting contraceptives, and self-efficacy statements. The “prompt” was an action the adolescent could take at that moment, such as a link to a family planning clinic website or website with contraception advice. “Prompts” were modeled as “Cues to action,” also part of the Health Belief Model. We iteratively developed 60 texts that included a “hook” and “prompt.” Examples of texts included the following:

ERdoc: All services are PRIVATE at the Family Planning Clinic. You don’t need a parent to receive care. (link to the local family planning clinic website)

ERdoc: Is NOW the right time to become pregnant? Talk to your partner. Or have him watch this. (link to Besider.org)

ERdoc: 1 Depo shot = 3 months of not having to worry about getting pregnant. Talk to your doctor. Learn now. (link to stayteen.org)

### Data Analysis

We sought IRB approval to conduct 20 interviews; however, interviews concluded sooner when saturation was reached such that no new themes emerged. Two investigators (LC and TH) coded the transcripts using Excel (Microsoft Corporation) and the qualitative software NVivo version 10 (QSR International), each independently generating a set of codes. A codebook was then developed and used to code the transcripts using a thematic analysis. Study team members discussed discrepancies in coding until consensus was achieved.

## Results

We conducted 14 interviews, and no new themes emerged after the twelfth interview. Participants were predominantly older adolescents, Hispanic, insured, and frequent ED users (Table 1).

All but one participant had an unlimited texting data plan, 79% (11/14) used passwords to protect their phones, and 71% (10/14) sent or received 30 or more texts per day. A summary of our themes, definitions, and exemplary quotes can be found in Table 2.

### Receptivity to Receiving Texts

#### Interested in Receiving Information via Texting Rather Than by Phone or Email

Texting was perceived as pervasive, easy, and informative. Participants noted often that “tech-heavy” teenagers do not listen to voicemails but “check the text messages incessantly.”

#### Considered Texts to Be Safe, Because They Are Confidential and Will Not Cause Embarrassment or Harm

Messages were perceived to be private, “something between me and the doctor.” Among participants who admitted that a parent or boyfriend might see their phone, it was felt that this person would be supportive, or it would be easy to deny knowing the sender.

#### Texts Sent by a Physician Are Trustworthy and Motivating

Participants showed interest in learning about “something new” from physicians. Messages from a trustworthy source seemed to drive ambition to go to a clinic or try a contraceptive.

### Preferred Content of Text Messages

#### Chose Messages That Contain Sexual Health Facts Not Previously Known to Them

Participants favored educational messages about different types of contraceptives and supported messages that dispelled misconceptions, such as the message, “Many contraception methods cause no weight gain.” The majority interviewed also stressed the importance of writing not only about pregnancy prevention but also sexually transmitted diseases, as one female affirmed that, even with the Depo shot, “you can still catch an infection.”

#### Emphasized the Importance of a Respectful Staff at Free and Confidential Clinics

There was a request for information about confidential clinics, their telephone numbers, and repeated directions to clinics, for those who are “too shy to call.” Clinics with walk-in policies were preferred, where you could simply “show up.”

#### Favored Messages Commenting on the Belief in One’s Capacity to Achieve a Behavior

Females responded favorably to messages about self-efficacy and “leading them in the right route.” They preferred reminders “to remember it’s your own life and you do what you want to do and you can say no (to sex without condoms) and that’s okay.”

#### Interested in Links to Videos, Quizzes, Websites, or Events

Most females said they would click on a link, especially when “sitting at home on your computer... (with) time your hands.” There was interest in sharing links with friends or boyfriends.

**Table 1:** Characteristics of female adolescent participants in the emergency department (ED) (n=14).

Characteristics	n (%)
Age (18-19 years)	10 (71%)
Hispanic ethnicity	13 (93%)
Insured	13 (93%)
In college	8 (57%)
Prior pregnancy	3 (21%)
Used any effective contraception in the past	11 (79%)
Currently in a sexual relationship	12 (86%)
Saw a primary care provider in the past year	14 (100%)
Received medical care in ED in the past year	13 (93%)

**Table 2:** Summary of codebook organizing themes, definitions, and exemplary quotes.

Theme		Definition	Quote
**Receptivity to texting from the ED**			
Preference		Interested in receiving information via text messages rather than by phone or email	It will be easier than email or calling on the phone because I might not get the phone or signal...I am always texting. [Participant #3]
Comfort		Considered text messages to be safe, because they are confidential and will Not cause embarrassment or harm	If my mom passes by while my phone is charging...and she sees that (text)...‘Oh Mom, it’s just the clinic. They have a new texting thing. Yeah Mom, come on. We’re not living in the 90s anymore’…She’d actually be very happy with that. [Participant #7]
MD-patient relationship		Text messages sent by a physician are trustworthy and motivating	I think she (older sister) would be comfortable with it because I am actually talking to a doctor, not like a regular person. [Participant #2]
**Preferred content of text messages**			
Information		Chose messages that contain sexual health facts not previously known to them	It’ll be good to write (about) STDs and stuff, because it’s not only about pregnancy. [Participant #1]
Services		Emphasized the importance of a respectful staff at free and confidential clinics	You have to make sure you tell them that their information is confidential. And that it’s free...because most of my friends don’t have insurance. [Participant #11]
Self-efficacy		Favored messages commenting on the belief in one’s capacity to achieve a behavior	You are responsible for your own body. [Participant #10]
Links		Interested in links to videos, quizzes, websites, or events	If you want more information, you can just click on the link. [Participant #13]
**Preferred text message structure**			
Word choice		Preferred simple, nonaccusatory words	If you choose to have unprotected sex, you are choosing to get pregnant...I think that one might be a little bit too accusatory...I don’t like being told what to do. [Participant #9]
Order		Messages should have a certain order and contain a header	Because sometimes we get those...random messages from like my phone company…And I just delete them. [Participant #6]
Customization		Commented on personalized text messages	Those girls...think they’re going to be with that boyfriend for the rest of their lives. And the boyfriend tells them “Oh, we don’t need to use condoms because it’s only me”...They don’t want to get pregnant because they have hopes and dreams...But then there is the totally different girl who’s sleeping with anyone. And she doesn’t want to get pregnant and she doesn’t use contraception either. So maybe those are two separate girls that you can have two separate sorts of text messages for. [Participant #14]
**Preferred schedule of text message delivery**			
Random versus selected times		Favored random timing of messages over selected time	I think definitely send them randomly for the element of surprise...if people are expecting it and know it’s coming, they going to like, ‘Oh, I know what this is. I don’t need it, delete it.’ [Participant #9]
Day		Reached no consensus on which days text messages should be sent	Maybe on the weekends, because usually the weekends are when mistakes happen. [Participant #11]
Time		Selected texts to be sent in the afternoon and early evening	Definitely not after 8 o’clock because that’s just creepy...after 8 o’clock is when the condom commercials come on, and sex...stuff like that. [Participant #7]
Frequency		Ranged in opinions of message delivery from twice a day to once a month	Don’t nag them like every day. [Participant #10]

### Preferred Text Message Structure

#### Preferred Simple, Nonaccusatory Words

Repeatedly, participants found texts with the word “you” to be offensive and condemning, such as, “Every minute another teenage girl becomes pregnant. Do you want to be one of them?” They preferred messages that provided information rather than made them feel as though they were being told what to do. In addition, they selected messages that were “direct and to the point,” much like facts on a “Snapple” bottle top. However, they also wanted “supporting detail” such as a link with additional trustworthy information. Importantly, they preferred the word “birth control” rather than “contraception” and rejected abbreviations, especially when English was not their first language.

#### Messages Should Have a Certain Order and Contain a Header

Females approved messages with a header statement, like “ERDoc.” This provided them with knowledge regarding the sender—“a reliable professional source.” While they preferred repetitive information, they also voiced that each message could continue where the last message left off, as if all the messages were part of a larger dialogue.

#### Commented on Personalized Messages

Finally, participants favored interactive, personalized messages. They liked the idea of being able to ask questions through texting and receiving answers such as saying they do not want birth control pills and receiving information on another option or requesting messages less or more often.

### Preferred Schedule of Text Message Delivery

#### Favored Random Timing of Messages Over Selected Time

Participants explained that the element of surprise would stimulate interest. However, one possible benefit of receiving the messages the same time every day was knowing that the texts were coming from a doctor, thus creating trust.

#### Reached No Consensus on Which Days Texts Should Be Sent

Some participants thought message delivery on a weekend was impactful because “that is when mistakes happen.” On the contrary, weekends were the time females were busy and thus might not pay attention to their phones.

#### Selected Texts to Be Sent in the Afternoon and Early Evening

Participants preferred receiving messages neither too early in the morning nor too late at night.

#### Ranged in Opinions of Message Delivery From Twice a Day to Once a Month

Females varied widely in their judgment of how often messages should be sent. Some perceived daily messages as a “nag,” whereas others viewed repetition beneficial in case they missed a prior text. In addition, messages sent weekly were similar to “sale emails,” which they might disregard as spam.

## Discussion

### Principal Findings

In this study, adolescent females in the ED were uniformly receptive to using text messaging to promote pregnancy prevention. Text messages were considered confidential and truthful when coming from a medical source. Adolescents preferred messages that supported their ability to achieve a positive behavior, provided repeated information about locations of confidential services, were both personalized and interactive, and provided accompanying links on sexual health information previously unknown to them. Preferred word choice was nonaccusatory and direct. Although there was no consensus on the timing and frequency of messaging, there was preference for the random delivery of messages in the afternoon and evening.

The use of texting interventions has shown promise as a means to deliver health information and change health behaviors among ED patients, including reducing binge drinking, increasing postED follow-up, and increasing medication adherence [13-15]. One study noted improved ability to contact adolescents regarding sexually transmitted diseases [16]. In a study focused on violence prevention, Ranney and colleagues found that adolescents supported ED-based preventive health texting interventions, with an emphasis on personalized and tailored messages [10].

Prior research of adolescents in the outpatient (nonED) setting has found similar text message preferences regarding content, structure, tone, and confidentiality [6,17]. Adolescents in our study and several prior studies favored messages that focused on future opportunities and promoted self-efficacy [18]. We found our adolescents favored messages that focused on what they could do to prevent pregnancy as well as ones that addressed perceived barriers to obtaining contraception. These preferences match the behavioral health literature in which messages focusing on engaging in a particular behavior (gain-frame) appear more effective in changing a preventive behavior than those highlighting the consequences of failing to engage in a behavior (loss-frame) [19]. Finally, some of the variability in responses we noted and those noted in earlier literature suggests the need for customized message delivery and content for certain subgroups of patients; this may increase receptivity and self-referential processing of information [10,20].

### Limitations

Limitations to our study include enrollment of a convenience sample of predominantly Hispanic patients. This study explored texting from the ED; we did not specifically compare acceptability of texting from the ED versus other health care sites. In addition, we did not ask in-depth questions to determine the specific processes necessary to create and implement interactive, tailored messages. Furthermore, while this study included females aged 14-19 years, all participants were 16 years and older; females aged 15 and younger may have different opinions. Finally, we interviewed only females. Adolescent males may have unique views regarding ED-based pregnancy prevention interventions and the use of text messaging for the ED; this is an area for future study.

### Conclusions

Adolescent female patients at high pregnancy risk are interested in ED-based pregnancy prevention provided by text messages. We identified key preferences for the content, frequency, and timing of texts, which can guide the design of future interventions in the ED.
